# Anifrolumab for refractory discoid lupus: Two case reports of successful outcomes in Saudi Arabia

**DOI:** 10.1097/MD.0000000000042518

**Published:** 2025-05-16

**Authors:** Roaa Aljohani

**Affiliations:** a Department of Medicine, College of Medicine, Taibah University, Medina, Saudi Arabia; b Department of Medicine, King Faisal Specialist Hospital and Research Centre, Medina, Saudi Arabia.

**Keywords:** anifrolumab, discoid lupus erythematosus, Saudi Arabia

## Abstract

**Rationale:**

Discoid lupus erythematosus (DLE) is a chronic, refractory condition causing visible scarring and significant psychological distress. Standard treatments often fail to provide adequate relief, necessitating new therapeutic options.

**Patient concerns:**

A 43-year-old woman with longstanding DLE experienced recurrent scalp lesions unresponsive to multiple therapies, including high-dose corticosteroids, which caused adverse effects. A 19-year-old woman with systemic lupus erythematosus presented with persistent discoid scalp lesions, disfigurement, and emotional distress despite standard treatments.

**Diagnoses:**

Both patients were diagnosed with refractory DLE, confirmed by clinical findings and skin biopsies.

**Interventions and outcomes:**

Both patients received monthly intravenous anifrolumab (300 mg) alongside hydroxychloroquine and methotrexate. Significant improvement was noted after the first dose, including reduced lesion severity, erythema, and itchiness, with visible hair regrowth. The first patient Cutaneous Lupus Erythematosus Disease Area and Severity Index-Activity score improved from 18 to 3, allowing complete corticosteroid discontinuation. The second patient Cutaneous Lupus Erythematosus Disease Area and Severity Index-Activity score decreased from 8 to 1. No adverse effects were reported.

**Lessons:**

These cases demonstrate anifrolumab potential as a treatment for refractory DLE, offering rapid and sustained improvement in disease severity and quality of life while reducing corticosteroid dependency. These findings suggest anifrolumab as a viable alternative for challenging DLE cases, warranting further research to confirm its efficacy and safety in larger populations.

## 1. Introduction

Cutaneous lupus erythematosus (CLE) manifests in up to 80% of individuals with systemic lupus erythematosus (SLE). However, CLE can also occur independently, without progression to systemic involvement or the presence of autoantibodies. CLE significantly impacts quality of life and is clinically heterogeneous in both severity and morphology. Discoid lupus erythematosus (DLE) is a chronic form of cutaneous lupus characterized by erythematous, scaly lesions that progress to atrophy, fibrosis, and hypo-and/or hyperpigmentation. Current treatments for DLE remain challenging, expensive, and not always effective.^[1]^

Anifrolumab is a monoclonal antibody that targets type I interferon receptors that are pivotal in the pathogenesis of SLE. It was approved in 2021 for adults with moderate to severe SLE, following the results of the phase II MUSE trial and the phase III TULIP-1 and TULIP-2 trials; it has shown promise in treating SLE, particularly for patients with refractory cutaneous symptoms.^[2–4]^ A pooled analysis of the TULIP studies showed significant improvement in patients with a baseline Cutaneous Lupus Erythematosus Disease Area and Severity Index-Activity (CLASI-A) score of ≥10 when treated with Anifrolumab compared to placebo.^[5]^ Notably, only a few case reports document anifrolumab effectiveness in treating discoid lupus. This report presents 2 cases of patients with refractory discoid lupus who achieved positive outcomes with anifrolumab, emphasizing its potential in treatment-resistant cases.

## 2. Consent for publication

Both patients consented to the publication and use of their images. Ethical approval from the local ethics committee was not required.

## 3. Case presentation

Two cases of discoid lupus, refractory to immunosuppressive therapies, responded successfully to Anifrolumab without any reported adverse effects.

### 3.1. Case report 1

**Figure 1.**
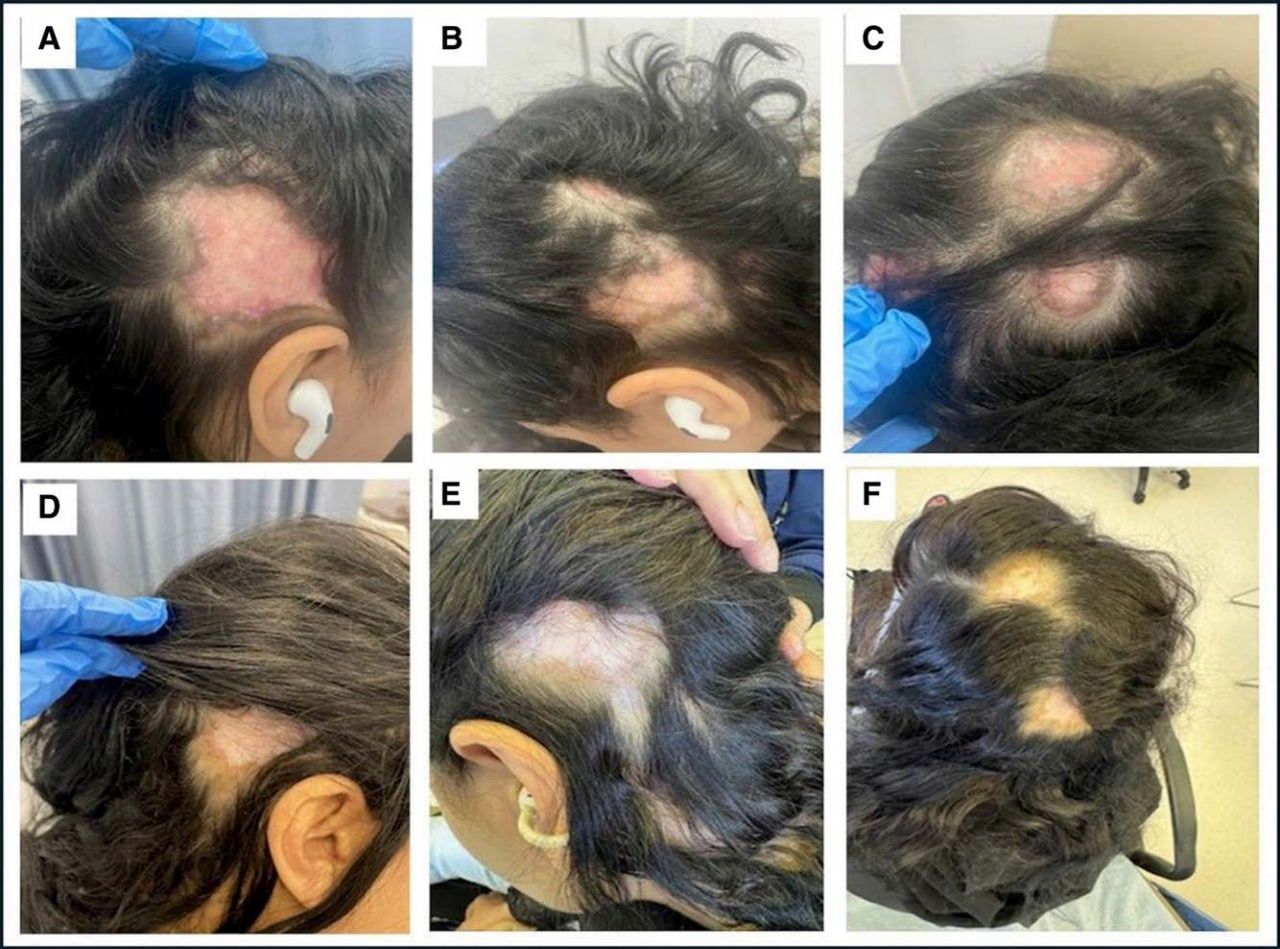
Discoid lesions in the first patient before and after anifrolumab treatment. Images (A, B, C) show erythematous, thickened, and hyperpigmented lesions with distinct scaling and inflammation, indicating active disease. Images (D, E, F), taken 3 months after anifrolumab therapy, demonstrate a marked reduction in erythema, thinning of lesions, decreased scaling, and improved pigmentation, suggesting a positive therapeutic response.

A 43-year-old woman known to have hypothyroidism was diagnosed with discoid lupus and patchy alopecia 5 years prior. Despite multiple treatments, including topical and intralesional steroids, hydroxychloroquine, oral corticosteroids, azathioprine, methotrexate, and belimumab, the patient experienced frequent relapses, particularly when corticosteroids were tapered. Consequently, she maintained a daily corticosteroid dose of 20 mg, which was the only effective treatment in controlling her symptoms. The patient presented with discoid lesions across multiple scalp areas, characterized by intense inflammation, redness, and hyperpigmentation. Her CLASI-A score was 18 (Fig. 1A–C). She reported significant itching and psychological distress due to the lesions’ appearance, though she denied any systemic symptoms associated with SLE.

Lupus serology showed a positive antinuclear antibody (ANA) by immunofluorescence with a titer of 1:80; however, subsequent tests were negative. Other autoimmune markers, including anti-double-stranded DNA and Smith antibodies, were also negative. Laboratory results, including complement levels, complete blood counts, inflammatory markers, and urinalysis, were within normal limits. Scalp skin biopsy findings were consistent with discoid lupus, showing epidermal atrophy, subepidermal bullous formation, vacuolar interface changes at the dermal-epidermal junction, and a thickened basement membrane. A superficial and deep perivascular and peri adnexal infiltrate was also identified.

The patient received intravenous anifrolumab at 300 mg every 4 weeks. Hydroxychloroquine and methotrexate were continued, and she was vaccinated against varicella-zoster 2 weeks prior to starting anifrolumab.

Following the first anifrolumab injection, the patient reported noticeable improvement. At the 3-month follow-up, there was a significant reduction in lesion inflammation, redness, and itchiness, with some hair regrowth in affected scalp areas (Fig. 1D–F). Her CLASI-A score decreased from 18 to 3 post treatment. Corticosteroids were tapered gradually until complete cessation, and the patient tolerated anifrolumab well, with no reported side effects or need for premedication before infusions. Continued follow-up over the subsequent 6 months revealed sustained remission, with no new flares or adverse effects. As of February 2025, the patient remains stable on anifrolumab, hydroxychloroquine, and low-dose methotrexate (7.5 mg weekly), with excellent disease control. The timeline from the patient initial clinical presentation to follow-up is summarized in Table 1.

**Table 1:** Clinical timeline for the first case.

Date	Clinical history	Intervention
August 2019	Active Discoid lesions in multiple scalp areas with patchy alopecia. Skin biopsy confirmed discoid lesions	Treated by dermatologist with intralesional and topical steroids
October 2019	No improvements. No systemic symptoms. ANA (1:80). Negative ENA. Other labs were normal	Started HQ. Azathioprine was introduced but caused leukopenia at higher doses and was discontinued after 3 months
January 2020	No improvement. The patient experienced depressive symptoms	Belimumab is given
October 2020	No improvement observed, and no additional tests conducted	Belimumab was stopped. Started oral corticosteroids (CS) at 60 mg
February 2023	The patient felt discouraged and continued self-administration of 20 mg CS	MTX 15 mg weekly added with a plan for slow steroid taper
August 2023	No change in discoid lesions. CLASI-A scored 18	Increased MTX to 20 mg and continued HQ. Patient declined tapering CS, maintaining 20 mg daily
April 2024	No response; unable to taper CS	Anifrolumab treatment started
July 2024	Significant clinical improvement observed. CLASI-A score decreased from 18 to 3	The patient received 3 doses of Anifrolumab along with MTX and HQ. Discontinued CS
February 2025	Patient has persistent improvement with no flares up, no side effects	On Anifrolumab, HQ, and MTX 7.5 mg weekly

ANA = antinuclear antibodies, CLASI-A = Cutaneous Lupus Erythematosus Disease Area and Severity Index-Activity, CS = corticosteroid, ENA = extractable nuclear antigen, HQ = hydroxychloroquine, MTX = methotrexate.

### 3.2. Case report 2

A 19-year-old woman with SLE was diagnosed one year earlier, based on a clinical history of arthritis, discoid lesions, leukopenia, and positive ANA titer. Initial treatment with corticosteroids and hydroxychloroquine resolved her arthritis but was less effective in managing the discoid scalp lesions. Upon presentation, she had active discoid lesions on multiple scalp areas, accompanied by patchy hair loss. She reported no history of systemic involvement.

**Figure 2.**
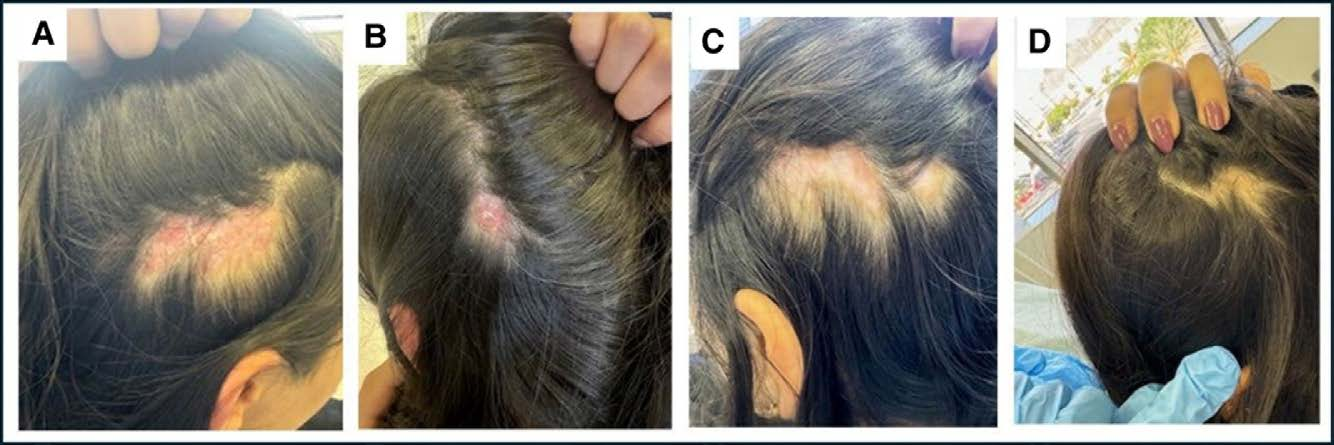
Discoid lupus lesions in the second patient before and after anifrolumab treatment. Images (A, B) taken before treatment show lesions with intense erythema, pronounced scaling, and raised edges, characteristic of active discoid lupus. After 3 months of anifrolumab therapy, images (C, D) demonstrate substantial improvements, including a marked reduction in erythema, diminished scaling, and decreased lesion elevation.

Physical examination confirmed active discoid lesions presenting as erythematous, atrophic patches with peripheral hyperpigmentation and patchy alopecia in various scalp regions (Fig. 2A, B). Her CLASI-A score is 8. Initial blood work revealed a low white blood cell count of 3.4 × 10⁹/L (4−11 × 10⁹/L), with normal renal and hepatic profiles and no proteinuria on urinalysis. ANA testing was positive via immunofluorescence at a titer of 1:160, while extractable nuclear antigen testing was negative. Complement testing initially showed a mildly low C3 level with normal C4 levels; however, subsequent tests returned normal results for all parameters.

The patient discoid lesions were initially treated with topical betamethasone and local corticosteroid injections, but these were ineffective. Azathioprine was prescribed for 5 months but also yielded no improvement. The patient declined high-dose corticosteroids; a 20 mg dose provided only a partial response and was stopped. Methotrexate was introduced at a maximum dose of 20 mg but was reduced to 15 mg due to gastrointestinal side effects, with no improvement in the lesions. Her CLASI-A score remained at 8 despite ongoing treatment with hydroxychloroquine and methotrexate. Ultimately, a decision was made to initiate monthly anifrolumab at 300 mg after a negative quantiferon screening. She received a varicella-zoster vaccine 2 weeks before starting anifrolumab, alongside continued hydroxychloroquine and methotrexate.

After initiating anifrolumab, the patient demonstrated marked improvement in cutaneous symptoms, with significant reductions in erythema and itching. Following the third injection, noticeable hair regrowth was observed, and the patient expressed satisfaction with the treatment outcome, reporting no adverse effects. Her CLASI-A score decreased from 8 to 1 (Fig. 2C, D). Continued follow-up over the next 5 months confirmed persistent clinical improvement with no disease flares or treatment-related adverse effects. As of March 2025, the patient remains stable on anifrolumab and hydroxychloroquine, with a reduced dose of methotrexate, and maintains excellent disease control. A summarized timeline of the patient clinical course is provided in Table 2.

**Table 2:** Clinical timeline for second case.

Date	Clinical data	Therapeutic intervention
July 2023	Arthritis, discoid lesions affecting 2 area of scalp. Leukopenia (3.4 X10^9/L). ANA (1:160). Low C3 (69 mg/dl). Other laboratory tests are normal including ENA	HQ, topical and intralesional CS
October 2023	No arthritis, persistent active discoid lesions. Normal complete blood counts, negative ANA, normal complements	Start her on CS 20 mg daily with Azathioprine
March 2024	No significant improvement in discoid lesions, no other symptoms	Discontinued Azathioprine. Started on MTX weekly
July 2024	Active discoid lesions persist, On methotrexate 20 mg weekly. CLASI-A score is 8	Anifrolumab is given alongside MTX and HQ
October 2024	Significant improvement after the third dose of Anifrolumab. CLASI-A score decreased from 8 to 1	Continued Anifrolumab, HQ, reduce dose of MTX
March 2025	No flares; patient has maintained clinical improvement without side effects	Continuing anifrolumab and HQ; MTX dose reduced

ANA = antinuclear antibodies, CLASI-A = Cutaneous Lupus Erythematosus Disease Area and Severity Index-Activity, CS = corticosteroid, ENA = extractable nuclear antigen, HQ = hydroxychloroquine, MTX = methotrexate.

## 4. Discussion

DLE is often a refractory condition that can severely impact patients’ quality of life, causing significant psychosocial stress due to visible scarring, especially in sensitive areas like the face and scalp. The chronic and frequently disfiguring nature of DLE lesions can lead to psychological distress, reduced self-esteem, and social withdrawal, as patients may feel self-conscious about their appearance. Effectively managing DLE is essential not only to control disease activity but also to alleviate its psychosocial burden and improve overall quality of life.

DLE treatment is challenging, as many patients do not respond to first-line therapies, such as topical corticosteroids and hydroxychloroquine. Limited treatment options exist beyond these initial therapies, often necessitating systemic immunosuppressive agents that carry additional risks and require careful monitoring. Furthermore, most available efficacy data for DLE treatments are derived from case reports or based on SLE management strategies, rather than controlled trials specific to DLE. This lack of dedicated research contributes to uncertainties in optimal management and underscores the need for targeted, evidence-based studies on effective therapies for refractory DLE cases.

Anifrolumab, a monoclonal antibody targeting the type I interferon receptor, has shown promise for the treatment of refractory DLE. Data from the TULIP trial demonstrated a significant improvement in skin disease activity, with reduced lesion severity in patients unresponsive to standard therapies.^[3,4]^ Although anifrolumab has shown effectiveness in clinical trials on cutaneous lupus, its effects on specific CLE subtypes are not well defined.^[2,4,5]^

Our patients showed a successful response to anifrolumab after multiple prior treatments had failed, with significant improvement after the first injection. Limited case reports have documented similar findings, consistently supporting the effectiveness of anifrolumab in refractory discoid lupus.^[6–10]^ Moreover, most cases reported improvement after early doses, highlighting the potential of anifrolumab for rapid and effective relief in difficult-to-treat cases.^[7,9,10]^ This suggests that anifrolumab is a potential option for managing DLE by effectively targeting the interferon pathway involved in lupus pathogenesis.

In conclusion, these cases underscore anifrolumab as a promising treatment option for refractory DLE, particularly when conventional therapies prove ineffective. Anifrolumab played a key role in reducing inflammation and lesion severity, improving quality of life by addressing both the physical and psychological burdens of DLE. These findings may guide physicians in considering anifrolumab as a viable option in challenging DLE cases. However, further research with larger studies is needed to confirm its efficacy and safety in this population, establishing a stronger basis for its use in managing resistant cases of discoid lupus.

## Author contributions

**Conceptualization:** Roaa Aljohani.

**Formal analysis:** Roaa Aljohani.

**Funding acquisition:** Roaa Aljohani.

**Writing – original draft:** Roaa Aljohani.

**Writing – review & editing:** Roaa Aljohani.

## References

[1] Verdelli A Corrà A Mariotti EB. An update on the management of refractory cutaneous lupus erythematosus. Front Med (Lausanne). 2022;9:941003. 36213629 10.3389/fmed.2022.941003 PMC9537468

[2] Furie R Khamashta M Merrill JT. Anifrolumab, an anti–interferon‐α receptor monoclonal antibody, in moderate‐to‐severe systemic lupus erythematosus. Arthritis Rheumatol. 2017;69:376–86. 28130918 10.1002/art.39962 PMC5299497

[3] Furie RA Morand EF Bruce IN.; TULIP-1 study investigators. Type I interferon inhibitor anifrolumab in active systemic lupus erythematosus (TULIP-1): a randomised, controlled, phase 3 trial. Lancet Rheumatol. 2019;1:e208–19. 38229377 10.1016/S2665-9913(19)30076-1

[4] Morand EF Furie R Tanaka Y.; TULIP-2 Trial Investigators. Trial of anifrolumab in active systemic lupus erythematosus. N Engl J Med. 2020;382:211–21. 31851795 10.1056/NEJMoa1912196

[5] Morand EF Furie RA Bruce IN. Efficacy of anifrolumab across organ domains in patients with moderate-to-severe systemic lupus erythematosus: a post-hoc analysis of pooled data from the TULIP-1 and TULIP-2 trials. Lancet Rheumatol. 2022;4:e282–92. 38288923 10.1016/S2665-9913(21)00317-9

[6] Bao A Petri MA Fava A Kang J. Case series of anifrolumab for treatment of cutaneous lupus erythematosus and lupus-related mucocutaneous manifestations in patients with SLE. Lupus Sci Med. 2023;10:e001007. 38114267 10.1136/lupus-2023-001007 PMC10748985

[7] Azuma N Natsuaki M Hashimoto N. Efficacy of anifrolumab in long-term intractable alopecia due to discoid lupus erythematosus. Mod Rheumatol Case Rep. 2024;8:267–71. 38597902 10.1093/mrcr/rxae018

[8] Shaw KS Rajeh A Le T. Anifrolumab for adolescent discoid lupus erythematosus. JAMA Netw Open. 2023;6:e2338200. 37851448 10.1001/jamanetworkopen.2023.38200 PMC10585408

[9] Kowalski EH Stolarczyk A Richardson CT. Successful treatment of severe chronic cutaneous lupus with anifrolumab: a series of 6 cases. JAAD Case Rep. 2023;37:21–9. 37324181 10.1016/j.jdcr.2023.04.024 PMC10265470

[10] Han S Ferrer J Bittar M Jones A. Alopecia secondary to severe discoid lupus responding to anifrolumab. Int J Womens Dermatol. 2023;9:e098. 37497192 10.1097/JW9.0000000000000098 PMC10368378

